# Metamifop as an estrogen-like chemical affects the pituitary-hypothalamic-gonadal (HPG) axis of female rice field eels (*Monopterus albus*)

**DOI:** 10.3389/fphys.2023.1088880

**Published:** 2023-01-19

**Authors:** Yi Zhang, Tianyu Guan, Long Wang, Xintong Ma, Chuankun Zhu, Hui Wang, Jiale Li

**Affiliations:** ^1^ School of Life Science, Huaiyin Normal University, Huai’an, China; ^2^ Key Laboratory of Freshwater Aquatic Genetic Resources Ministry of Agriculture, Shanghai Ocean University, Shanghai, China

**Keywords:** *Monopterus albus*, metamifop, endocrine disruption, hypothalamic-pituitary-gonadal axis, sexual reversal

## Abstract

Metamifop (MET) is a widely used herbicide. It is likely for it to enter water environment when utilized, thus potential impacts may be produced on aquatic animals. Little information is available about its effects on the endocrine system of fish to date. In the current study, female rice field eels (*Monopterus albus*) were exposed to different MET concentrations (0, 0.2, 0.4, 0.6, 0.8 mg L −1) for 96 h to examine the effect of MET on the hypothalamic-pituitary-gonadal (HPG) axis and sexual reversal. The results showed that high concentrations of MET exposure increased vitellogenin (VTG) levels in liver and plasma, but plasma sex hormone levels were not affected by MET exposure. MET exposure increased the expression of CYP19A1b and CYP17 that regulate sex hormone production in the brain, but the expression of genes (CYP19A1a, CYP17, FSHR, LHCGR, hsd11b2, 3β-HSD) associated with sex hormone secretion in the ovary and the estrogen receptor genes (esr1, esr2a, esr2b) in the liver were all suppressed. In addition, the expression of sex-related gene (Dmrt1) was suppressed. This study revealed for the first time that MET has estrogen-like effects and has a strong interference with the expression of HPG axis genes. MET did not show the ability to promote the sexual reversal in *M. albus*, on the contrary, the genes expression showed that the occurrence of male pathway was inhibited.

## Introduction

Endocrine-disrupting chemicals (EDCs) can interfere with various aspects of biological endocrine secretion through intake, accumulation and other ways. Exposure to EDCs are thought to have an irreversible effect on animals during critically susceptible phases of sexual differentiation. Synthetic chemicals particularly used in agriculture are thought to cause endocrine disturbances in wildlife, and herbicides are among the most commonly used agricultural chemicals (Mnif et al., 2011; Combarnous, 2017). For a long time, herbicides have been widely concerned because of their behavioral toxicity (Yalsuyi et al., 2021), neurotoxicity (Merola et al., 2022), reproductive toxicity (Soni and Verma, 2020) and many other effects on aquatic organisms.

Aryloxyphenoxypropionate (AOPP) herbicides have been widely used in paddy fields due to their excellent weed control ability. Metamifop (MET) ((R)-2-[4-(6-chloro-1,3-benzoxazol-2-yloxy) phenoxy]-2′-fluoro-N-methyl propionanilide), an AOPP herbicide developed by Dongbu Hannong Chemical Co., Ltd (South Korea), can effectively control annual weeds by inhibiting the activity of acetyl-CoA carboxylase (ACCase), thereby interfering with lipid synthesis (Xia et al., 2016). Due to its high safety to cereal crops, MET has been widely used around the world, including China, Korea, Japan, Philippines (Parker et al., 2015; Cooper et al., 2016). The half-life of MET varies in different environments, for example, 15.83–17.5 days in laboratory water (pH = 7.0) (Saha et al., 2018), 21.5–40.80 days in paddy fields (Wang et al., 2012), and about 70 days in soil (Janaki and Chinnusamy, 2012). Because MET residues persist in water and soil for a longer time, the implications of MET for aquatic organisms should be concerned.

MET was reported to be highly toxic to zebrafish (*Danio rerio*) embryos (acute 96 h LC_50_ value of 0.648 mg L^−1^) and juvenile zebrafish (acute 96 h LC_50_ value of 0.216 mg L^−1^) (Zhao et al., 2019). According to the information provided by the International Union of Pure and Applied Chemistry (IUPAC, 2009), MET is moderately toxic to rainbow trout (*Oncorhynchus mykiss*) (96 h LC_50_ value of 0.307 mg L^−1^), invertebrate (*Daphnia magna*) (48 h EC_50_ value of 0.288 mg L^−1^), and algae (72 h EC_50_ value of 2.03 mg L^−1^). A study of quizalofop-p-ethyl, also an AOPP herbicide, showed that exposure at sublethal doses increased estrogen axis activity in male zebrafish (Zhu et al., 2017). Recently, Liu et al. (2021) reported that the thyroid hormone levels and related genes expression in tadpoles (*Xenopus laevis*) were affected by MET, reflecting the endocrine interference effect of MET. To date, the impact of MET on the sex hormone of fish has not been reported, and the underlying mechanism is unclear.

*M. albus* is a commercially important freshwater species in China and very popular for its delicious taste. Although the prodution of *M. albus* in China was about 307,200 tons in 2020 (China Fisheries Statistical Yearbook, 2021), the supply still demand exceeds supply. It is difficult to meet the market demand due to the low yield of monoculture. In order to meet the demand and increase the income of farmers, the rice-eel farming model has been gradually promoted in recent years. In this case, use of herbicides in paddy fields is necessary to ensure yield, meanwhile the safety of herbicides for *M. albus* need to be considered. All *M. albus* are females prior to maturity and can naturally transform into males through a bisexual stage (Cheng et al., 2021). The low number of male *M. albus* is an important factor that restricts the artificial reproduction of *M. albus*. Therefore, normal sex reversal is crucial to the breeding of *M. albus*. Our preliminary experiment showed that sublethal concentration of MET (0.8 mg L^−1^) resulted in a significant increase in plasma vitellogenin (VTG) levels in female *M. albus* after 4 days of exposure. VTG has been shown to be a biomarker for EDCs (Yamamoto et al., 2017; Li et al., 2018; Merola et al., 2021). Therefore, MET may have an effect on endocrine homeostasis in *M. albus*, acting as an endocrine disruptor to affect sex reversal of *M. albus*. In the present study, the levels of plasma steroid hormone and the expression of related genes on hypothalamic-pituitary-gonadal (HPG) axis were detected to evaluate influence of MET on the endocrine system of *M. albus.* The results of this work may help elucidate the mechanism of MET action on the endocrine secretion and sex reversal of *M. albus*.

## Materials and methods

### Chemical

Metamifop (CAS No.256412-89-2, purity 99%) was supplied by Hubei JiangMinTaiHua Chemical Co., Ltd. (Hubei, China). MET stock solution of 1,000 mg L^−1^ was prepared as previously described by Zhao et al. (2019). Acetone containing 1% Tween-80 (v/v) was used to prepare solvent control in all the tests. For other chemicals, Analytical Reagent (AR) grade chemicals were used.

### Exposure experiment

Healthy female *M. albus* with ovary developing at the third stage (weight 10–15 g, length 24–28 cm) were taken from Luoma Lake, Jiangsu Province, China. *M. albus* were acclimated in polyethylene tanks (5 m × 0.8 m × 0.4 m, water depth 0.3 m) of the Jiangsu Special Aquatic Breeding Engineering Laboratory for 1 week. During the acclimation period, the eels were fed with earthworm (once a day, 19: 30 pm, approx. 1.5 g fish^−1^ day^−1^), with a 14 L: 10 D photoperiod. pH 7.8 ± 0.1; dissolved oxygen 9.0 ± 0.5 mg L^−1^; temperature 20°C ± 2°C; only exist the aerator working sound. Water was changed every other day. Feeding stopped 1 day before the experiment, and no feeding was given during all the experiment period. According to the results of preliminary experiment, the 96 h LC_50_ of MET to *M. albus* was determined to be 0.785 mg L^−1^. Dechlorinated tap water and 0.08% acetone and 0.0008% Tween 80 dissolved in dechlorinated tap water (v/v) were used as blank and solvent control, respectively. The concentration of MET in the experiment was set at 0.2, 0.4, 0.6, 0.8 mg L^−1^, respectively, and was determined using high phase liquid chromatography (HPLC). During the experiment, 144 *M. albus* were selected randomly into six groups, each group with three replications. Then the *M. albus* were placed in culture tanks (40 cm × 30 cm × 15 cm, water depth: 10 cm). The water tanks used in the experiment were covered with gauze to prevent eels from escaping, and the physicochemical parameters of the water were consistent with those in acclimation period. Tiles were placed as shelter. The test solutions were renewed every 24 h in order to maintain MET concentration constant and dead fish were cleared at the same time.

### Sample collection

After 96 h, eels were anesthetized with 3-aminobenzoic acid ethyl ester methanesulfonate (MS-222) (Sigma-Aldrich Co., St. Louis, MO, United States of America). The blood samples were collected by severing the caudal peduncle. The blood was immediately mixed with anticoagulant (0.50‰ EDTA) and centrifuge at 1,500 rpm/min for 30 min to obtain plasma. Then the plasma was extracted and stored at 4°C for analysis. After, the *M. albus* were decapitated on ice and ovary, brain (complex of brain and pituitary) and liver tissues were collected and preserved at −80°C.

### Hormone and vitellogenin measurement

Estradiol (E2), testosterone (T) and vitellogenin (VTG) in anticoagulated plasma and liver were detected by enzyme linked immunosorbent assay (ELISA), ELISA kits from Shanghai QiaoDu Biotechnology Co., Ltd. (ShangHai, China) were used following the instructions of the manufacturer.

### Gene expression

Total RNA of ovaries, brain and liver samples was extracted using TRIzol reagent (CWBIO, Beijing, China). The purity, concentration and integrity of RNA samples were detected by Nanodrop, Qubit 2.0, Aglient 2,100, respectively. Single-stranded cDNA was synthesized using HiFiScript cDNA Syntheses Kit (ComWin Biotech Co., Ltd, Beijing, China) following the instructions. The transcripts of genes were measured by LightCycler^®^ 480 II RT–qPCR machine (Roche, Switzerland). The primers were designed by Primer Premier 6.0 and are shown in Table 1. The reaction mixtures were 20 μL, containing 10 μL SYBR Premix Ex Taq (2 ×), primers (0.5 μM) 1 μL each, 2 μL cDNA sample, and 6 μL dH_2_O. Cycling conditions were 95°C for 5 min, followed by 40 cycles of 95°C for 10 s, 60°C for 30 s and 72°C for 30 s. All samples were run in triplicate, and each assay was repeated three times. The threshold cycle (Ct) values were obtained from each sample. Relative gene expression levels were evaluated using 2^−△△CT^ method (Livak and Schmittgen, 2001).

**TABLE 1:** The sequences of primers used in this experiment. All sequences are shown 5’→ 3’.

Target gene	Forward primer
esr1	F: CACACAAGCACACCCAACAGG
	R: CGCTCAGCCGTCTTAGTTCATA
esr2a	F: AAATCTAGGGTCTTTCCCGTTG
	R: GCCTTTTCCGCATCAGACAG
esr2b	F: TCTCCCACATCCGCCAAGT
	R: CAGAGGTCAAAGGCAAATCCA
CYP19A1a	F: AAAATGCTCCTCGCCGTTAC
	R: TCACCATGGCAATGTGCTTG
CYP19A1b	F: AGAGTTGAAGATAGTGGAGGAG
	R: CACTATATTTCAGAGCTGACTGG
CYP17	F: GGTCCCCCAAGCTTAGTGAC
	R: AGATAGCTGGGTGATGGGGT
FSHβ	F: GAGAGAGAATTCTGCAGCTTCAGCTGTCATCCA
	R: GAGAGACTCGAGAGGGAGGGGCTCACAGTA
LHβ	F: GAGACCATGGGCTTCCAGCTGCCGCCCTGCC
	R: GAGAGAGGATCCTTAGTAGTAGAAAGGTATGTC
FSHR	F: CCCATTGTTGGGGTCAGCAGCTACA
	R: GCAAATGAAGTCAGTGAAGATAAGG
LHCGR	F: AGGAAGGATGTGTGTCTGTTGC
	R: TCTGTGTTCGTGGTCATGTGG
hsd11b2	F: ATGAAACCCAAAGTGAACCAGA
	R: AAGACGAAGGGGCTCAAAGA
3β-HSD	F: GATGGGACCAAACTCCAGGG
	R: ACGCATATGGCCCAAAAGGA
Dmrt1	F: TCTCAGTACCGCATGCATTC
	R: TGTTGTTGTTGCTGCTGCTG
foxl3	F: CATCATCTCCAAGTTCCCCTACTAC
	R: GGTCTGTAGGGTCTCCTAACTCTCTT
JNK1	F: GTGCTCATGAAATGTGTCAACCA
	R: GGACAGTCTCTCGTGATCCAGC
β-acting	F: TCAACACGCCTGCCATGTAT
	R: CGCTCAGCTGTGGTAGTGAA

### Statistical analysis

Prior to conducting statistical comparisons, the experimental data which were presented as the mean ± standard deviation were assessed for normality and homogeneity of variances using the Shapiro-Wilk test and Levene’s test, respectively. The experimental data were analyzed using unifactor ANOVA, followed by SNK *post hoc* test. The critical value for statistical significance was *p* < 0.05. All statistical analyses were conducted using SPSS 26.0 software (SPSS, Chicago, IL).

## Results

### Solvent effect and analytical quantification of exposure solutions

Statistical analysis showed that solvent had no effect on all indicators in this study. Therefore, the solvent group could be used as a control. HPLE analysis of the water samples indicated that the exposure solutions ranged approximately 93–120% of all nominal concentrations (Table 2). Since all tested solutions were updated daily and the concentration of solutions were measured before and after the replacement, therefore, the nominal dose can represent the actual content in this study.

**TABLE 2:** Actual metamifop concentration (mg L ^−1^ ± SD) in the water during the experiment.

Actual concentration	Nominal metamifop concentration (mg L^−1^) ± SD			
	0.2	0.4	0.6	0.8
T0	0.24 ± 0.02	0.43 ± 0.04	0.66 ± 0.02	0.85 ± 0.01
T24	0.19 ± 0.01	0.38 ± 0.04	0.56 ± 0.05	0.76 ± 0.03

SD: standard deviations.

T0: at the beginning of each renewal of test solution.

T24: 24 h after each renewal of test solution.

### Effect of metamifop on sex hormones

**FIGURE 1:**
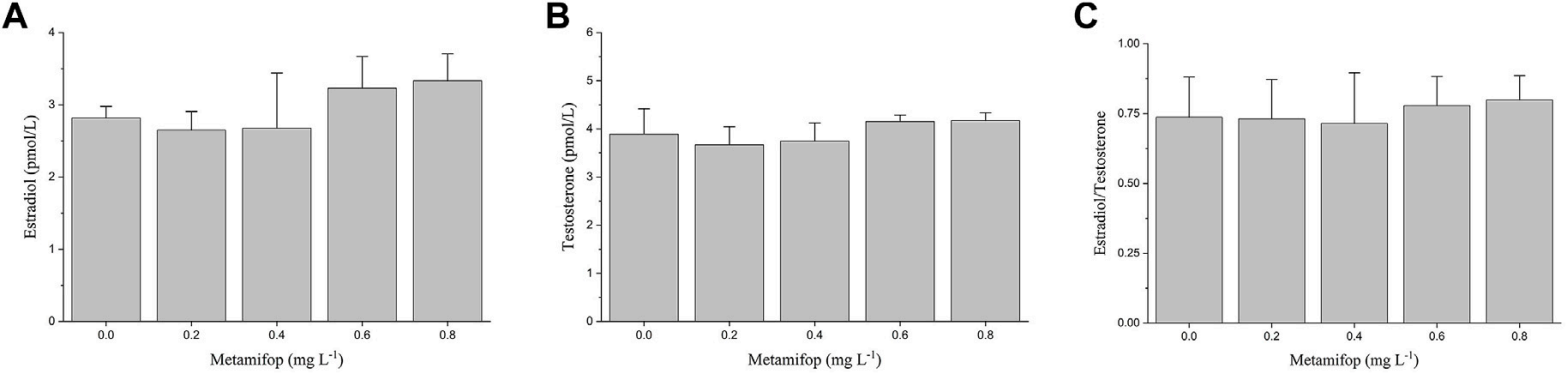
Plasma E2 **(A)**, T **(B)** levels and E2/T ratio **(C)** in female *Monopterus albus* exposed to 0, 0.2, 0.4, 0.6, 0.8 mg L^−1^ metamifop for 96 h. Results are presented as mean ± SD of three replicate samples (*n* = 8). *p < 0.05 and **p < 0.01 indicate significant differences between exposure and control group.

In the present study, no significant effect on measured plasma E2 and T levels were observed at all concentrations of metamifop (*p* > 0.05). However, compared with the control group, E2 and T showed a non-significant decrease at 0.2 mg L^−1^ and 0.4 mg L^−1^ of MET and a non-significant increase at 0.6 mg L^−1^ and 0.8 mg L^−1^ of MET (Figures 1A,B). In addition, the ratio of estrogen to androgen (E2/T) remained unchanged after exposure to MET (Figure 1C).

### Effect of metamifop on vitellogenin

**FIGURE 2:**
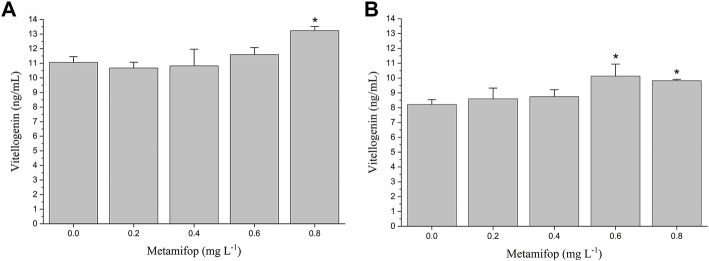
Plasma vitellogenin (VTG) **(A)** and liver VTG **(B)** levels in female *Monopterus albus* exposed to 0, 0.2, 0.4, 0.6 and 0.8 mg L^−1^ metamifop for 96 h. Results are presented as mean ± SD of three replicate samples (*n* = 8). *p < 0.05 and **p < 0.01 indicate significant differences between exposure and control group.

The VTG content in plasma of female *M. albus* was significantly elevated (*p* < 0.05) in the 0.8 mg L^−1^ treatment compared with the control (Figure 2A). In liver, VTG levels were significantly increased (*p* < 0.05) in both 0.6 and 0.8 mg L^−1^ MET-treated groups than the control group (Figure 2B). However, compared with the control group, at lower MET concentrations (0.2 mg L^−1^ and 0.4 mg L^−1^), VTG levels in both plasma and liver did not change significantly.

### Effect of metamifop on the expression of endocrine-related genes

#### Endocrine-related gene expression in the brain

**FIGURE 3:**
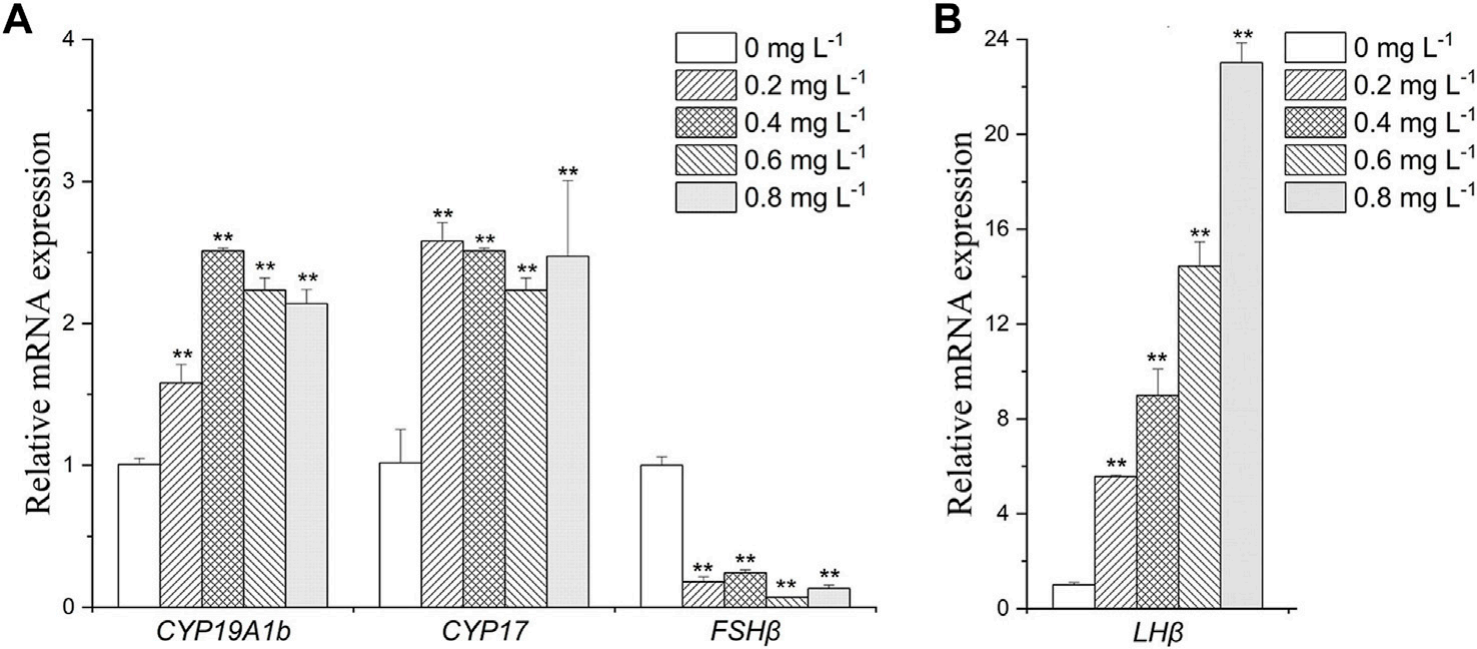
Expression profiles of genes **(A)**: *CYP19A1b*, *CYP17*, *FSHβ*; **(B)** *LHβ*) in *Monopterus albus* brain exposed to 0, 0.2, 0.4, 0.6 and 0.8 mg L^−1^ metamifop for 96 h. Results are presented as mean ± SD of three replicate samples (*n* = 8). *p < 0.05 and **p < 0.01 indicate significant differences between exposure and control group.

In brain, *CYP19A1b* was upregulated in MET exposure groups compared with the control (by 1.6- to 2.5-folds) (Figure 3A). *CYP17* expression was increased by about 2- to 2.5- folds in all exposure groups (Figure 3A). *FSHβ* was downregulated in all exposure groups (by 2.5- to 5-folds) (Figure 3A). The expression of *LHβ* was increased by 5.5-fold, 9.0-fold, 14.0-fold and 23.0-fold after exposure to 0.2, 0.4, 0.6, and 0.8 mg L^−1^ MET, respectively (Figure 3B).

#### Endocrine-related gene expression in the ovary

**FIGURE 4:**
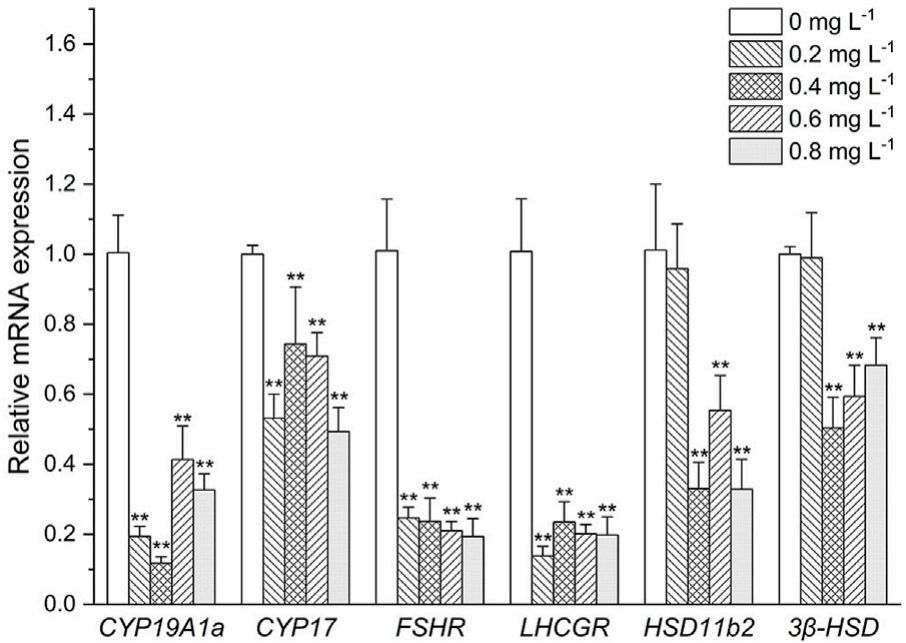
Expression profiles of genes associated with the HPG axis in *Monopterus albus* ovary exposed to 0, 0.2, 0.4, 0.6, and 0.8 mg L^−1^ metamifop for 96 h. Results are presented as mean ± SD of three replicate samples (*n* = 8). *p < 0.05 and **p < 0.01 indicate significant differences between exposure and control group.

As shown in Figure 4. The expressive levels of *CYP19A1a* and *CYP17* were significantly decreased in all the MET exposed groups compared with the control (*p* < 0.05). Similarly, *FSHR* and *LHCGR* mRNA levels were significantly decreased at all MET concentrations compared to the control (all about 5-fold). At low MET concentration (0.2 mg L^−1^), both the expressive of *HSD11b2* and *3β-HSD* did not occur significant change. However, after the increase of MET concentration, the expressive levels of *HSD11b2* and *3β-HSD* were decreased. For example, the expressive level of *HSD11b2* was significantly decreased when exposure to 0.4, 0.6, and 0.8 mg L^−1^ MET (3.3-fold, 2-fold and 3.3-fold, respectively). Transcription level of *3β-HSD* was downregulated by 2-fold in the 0.4 mg/L group (*p* < 0.05), 0.6-fold in the 0.6 mg L^−1^ group (*p* < 0.05) and 0.7-fold in the 0.8 mg L^−1^ group (*p* < 0.05).

#### Endocrine-related gene expression in the liver

**FIGURE 5:**
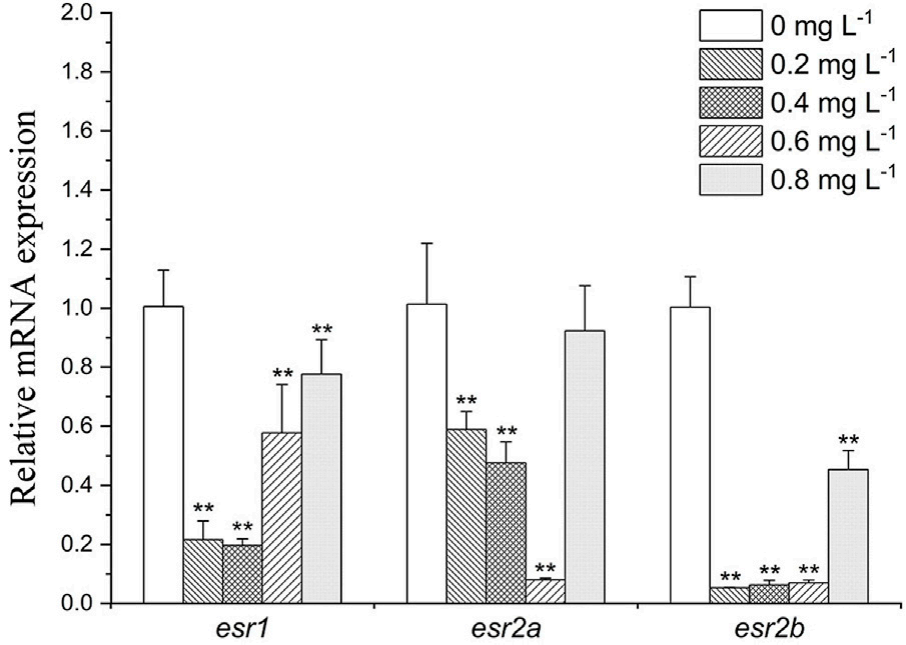
Estrogen receptor gene expression profiles in *Monopterus albus* liver exposed to 0, 0.2, 0.4, 0.6, and 0.8 mg L^−1^ metamifop for 96 h. Results are presented as mean ± SD of three replicate samples (*n* = 8). *p < 0.05 and **p < 0.01 indicate significant differences between exposure and control group.

As shown in Figure 5, in liver, *esr1*, *esr2a*, *and esr2b* were downregulated in all exposure groups (by 1.3- to 5-folds, 1.1- to 5-folds and 2- to 10-folds, respectively). While under the influence of high concentration of MET (0.8 mg L^−1^), the expression of the receptor genes *esr1*, *esr2a*, and *esr2b* was recovered compared to other treatment groups.

### Effect of metamifop on the expression of sex-related genes

**FIGURE 6:**
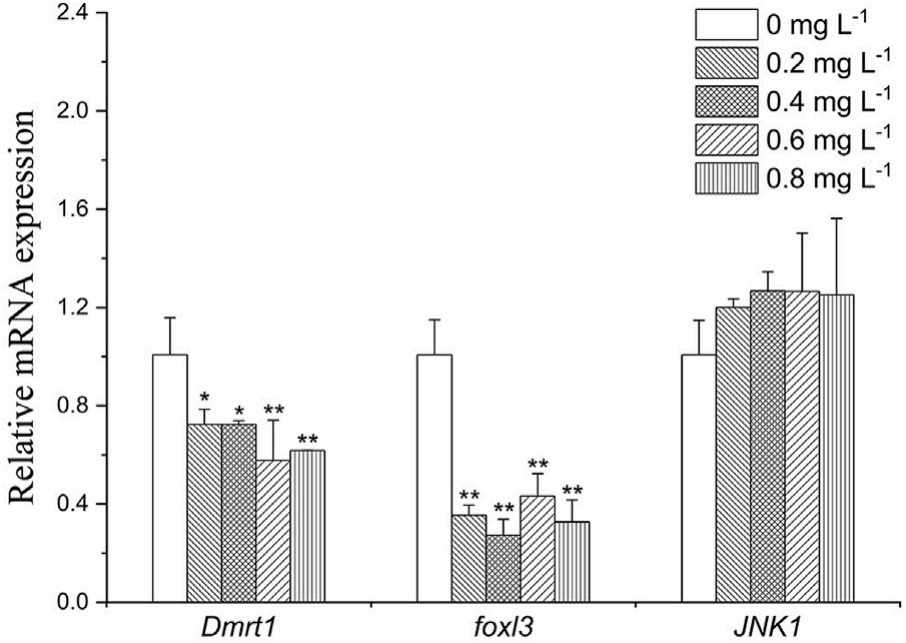
Sex-related gene expression profiles in *Monopterus albus* ovary exposed to 0, 0.2, 0.4, 0.6, and 0.8 mg L^−1^ metamifop for 96 h. Results are presented as mean ± SD of three replicate samples (*n* = 8). *p < 0.05 and **p < 0.01 indicate significant differences between exposure and control group.

As shown in Figure 6, *Dmrt1* expression decreased by about 0.7-fold in the 0.2 and 0.4 mg L^−1^ group (*p* < 0.05), and 0.6-fold in the 0.6 mg L^−1^ group (*p* < 0.05). *foxl3* was downregulated in all exposure groups (by about 2.5- to 3.3- folds). Expression level of *JNK1* was slightly increased after exposure to MET (by about 1.2- to 1.3- folds).

## Discussion

Vitellogenin (VTG), the precursor of yolk proteins, is produced after estrogen combine with specific Estrogen receptors (ESRs) in the liver, while VTG is absent or present at very low concentration in male and juvenile fish (Tyler et al., 1996). It has been found that some endocrine disruptors have estrogen-like effect and can induce the synthesis of VTG (Nikoleris et al., 2016). Therefore, VTG is considered as a biomarker of the presence of environmental estrogen-like compounds. In the present study, the upregulation of VTG concentrations in liver and plasma of female *M. albus* was observed at high concentration of MET exposure (Figure 2), while no significant change of E2 was found at all MET concentrations. This indicates that MET may have a potential estrogenic effects, mimicking the effect of endogenous estrogen and directly acting on liver estrogen receptors and stimulating VTG synthesis.

Sex steroids exist naturally in vertebrates, it can affect the sex differentiation and reproduction of fish by regulating various parts of the HPG axis (Li et al., 2013). At present, it is found that some exogenous molecules can affect sex steroids by acting on key steroidogenic enzymes or eliminating endogenous hormones (Ankley et al., 2005; Cheshenko et al., 2008). Therefore, the measurement of sex steroid hormones is often suggested as one of the most integrative and functional endpoints to understand the effects of external chemical on sexual development in fish (Ma et al., 2012; Jo et al., 2014). Unlike previous endocrine disruptors that affect the secretion of sex steroids, in the present study, plasma E2 and T levels of *M. albus* showed no significant change under the influence of MET (Figures 1A,B). This indicates that the interference effect of MET on *M. albus* is direct, not through affecting endogenous sex hormones, and then regulating the genes expression on HPG axis through the changing sex hormone levels. Since the ratio of E2/T is thought to regulate the direction of sexual differentiation in fish (Rougeot et al., 2007; de Carvalho et al., 2017), the difference in E2/T ratio may determine whether the gonad develops into ovary or testis. Previous studies also showed that sex reversal in *M. albus* was accompanied by a significant increase in androgens (Chan and Phillips, 1969). However, under the influence of MET, the sex hormone level of *M. albus* did not change significantly. This indicates that the sexual reversal of *M. albus* may not be affected by exposure to MET because of the consistency in sex hormone.

However, sex steroid synthesis and homeostasis depend on regulation of the HPG axis (Nagahama, 1994), which involves the actions of several genes. As the last enzyme in the steroidogenic pathway, cytochrome P450 aromatase (CYP19s) is in charge of turning androgens into estrogens, which has a direct impact on the E2/T ratio. There are two genes in teleost, *CYP19A1a* and *CYP19A1b*, which encode two proteins with different structures but the same catalytic activities (Kazeto et al., 2004). The *CYP19A1a* is predominantly expressed in ovary, and *CYP19A1b* predominantly expressed in brain. As *CYP19A1b* has estrogen response elements (EREs) (Callard et al., 2001), chemicals with estrogenic effects may induce structural changes of receptor proteins by binding to estrogen receptors. This results in dimerization and binding with EREs sequence, inducing *CYP19A1b* expression (Zhu et al., 2017). However, EREs are not present in all teleosts’ *CYP19A1a* promoter sequences (Piferrer and Blazquez, 2005), which result in the response of *CYP19A1a* expression to estrogen or estrogenic EDCs that vary in different fish (Li et al., 2013). In the present study, the mRNA expression of *CYP19A1b* was significantly upregulated, but the mRNA expression of *CYP19A1a* was significantly downregulated. This indicates that MET may be used as a ligand to simulate the direct binding of endogenous estrogen to estrogen receptor and estrogen receptor on *CYP19a1b* to induce the expression of *CYP19a1b*. Faria et al. (2021) also found that fenitrothion, a pesticide, could increase the expression of *CYP19a1b* in zebrafish larvae by acting on sex receptors. CYP17, an enzyme that synthesizes androgens in vertebrates (Govoroun et al., 2001; Chen et al., 2010), is found to be expressed primarily in brain and gonad (Chen et al., 2010). In this study, the *CYP17* expression showed synchrony with that of *CYP19*, and was increased in brain and decreased in ovary under the influence of MET. The contrary respect at down-regulations of *CYP19A1b* and *CYP17* in brain and transcriptional upregulation of *CYP19A1a* and *CYP17* in ovary may explain, the secretion of sex hormones in the brain and ovary may have different response mechanisms after Met stress, which may be the reason why the concentrations of E2 and T in female plasma remained constant. In addition, the difference in the expression in ovary and brain suggests that MET have different interference mechanisms in different tissues.

In fish, the gonadotropins follicle stimulating hormone (FSH) and luteinizing hormone (LH) are secreted by the pituitary and act by binding to specific receptor (FSHR, LHCGR) in gonad, which are the primary mediators of signals to induce steroidogenesis and gametogenesis (Clelland and Peng, 2009). FSH and LH are heterodimeric glycoproteins consisting of a common α-subunit and unique β-subunits, FSHβ and LHβ, respectively (Pierce and Parsons, 1981). The effects of exogenous hormones on FSH and LH mainly act on the mRNA levels of the corresponding β subunits (Li et al., 2013). There exist both positive and negative feedback of steroids for regulation of gonadotropin in fish. Positive feedback of sex steroid is mainly through direct interaction of the ESRs complex with an estrogen regulatory element on *FSHβ* and *LHβ* genes. Negative feedback of sex steroid on gonadotropin mainly includes two: one is reducing the content of gonadotropin releasing hormone (GnRH) receptor on the pituitary to reduce the regulation from GnRH (Habibi et al., 1989), the other is to act directly on GnRH neurons in hypothalamus or other neural systems known to affect GnRH system, regulating the release of more or less GnRH from the hypothalamus, to Regulates the release of gonadotropins from the pituitary gland (Montero et al., 1995). In our case, after expose to MET, *FSHβ* expression was suppressed, while *LHβ* expression was activated. The results were similar to the effect of E2 on the expression of *FSHβ* and *LHβ* in the pituitary cells of *M*. *albus* cultured *in vitro* (Zhang et al., 2014). This indicates that the effect of estrogen on the expression of *FSHβ* may be mainly through GnRH system, while the effect on the expression of *LHβ* is direct. The differential expression of *FSHβ* and *LHβ* in *M. albus* indicates that the MET may has the same biological effects as E2. In addition, it has been shown that gonadotropin signaling may be involved in the sex changes in a variety of teleost fish, such as *Trimma okinawae* (Kobayashi et al., 2009) and *Epinephelus merra* (Kobayashi et al., 2010). Zhang et al. (2010) also found that the ratio of *FSHβ*/*LHβ* was important for the gonadal differentiation of *M. albus*. However, it is unclear whether the decline in the ratios of *FSHβ*/*LHβ* after MET exposure is related to sexual reversal of *M. albus*, which needs further research. FSH and LH eventually act on gonads through blood circulation to promote the release of gonadal hormones. As gonadotropin receptors, the expression of *FSHR* and *LHCGR* should be closely related to the changes of FSH and LH. In this study, the expression levels of *FSHR* and *FSHβ* were consistent while the expression levels of *LHCGR* and *LHβ* was opposite under the influence of MET. The processes for synthesis and release of LH have been proven to be different, synthesized LH was stored in the pituitary gland rather than released into the plasma (Mateos et al., 2002). Thus, the expression of *LHCGR* in ovary is not directly affected by *LHβ* expression, the expression of *LHCGR* may be more influenced by LH in plasma.

In teleost, the type 2, 11β-hydroxysteroid dehydrogenase (*HSD11b*_*2*_) and 3β-hydroxysteroid dehydrogenase (*3β-HSD*) are involved in androgen synthesis, which are considered to play an important role in masculinity (Kusakabe et al., 2003; Ma et al., 2012). In the present study, *HSD11b2* and *3β-HSD* expression decreased significantly after MET exposure. Androgen synthesis genes can be suppressed by estrogen-like chemicals, which has been reported in *odontesthes bonariensis* (Perez et al., 2012), *Oncorhynchus mykiss* (Nakamura et al., 2009) and *Salmo salar* (Kortner et al., 2009)*.* The results of the present study were similar to these previous results, which further demonstrate that MET may have estrogenic potency. However, unlike previous studies, plasma T levels did not decrease due to the decreased expression levels of androgen synthesis genes. One explanation for this phenomenon is that production of steroids is a complex process with multiple sensitive control points, so the genes within the steroidogenesis pathway are not transcribed to the same extent and have no simple linear relationship with hormone production. However, whether this reduction in the expression of genes associated with sex steroid levels inhibits the reversal of female *M. albus* to males requires further investigation.

ESRs are ligand-activated transcription factor that interacts with the response elements in the target gene promoter to activate or inhibit transcription (Heldring et al., 2007). Both estrogens and estrogen-like environmental chemicals can interact with the ligand binding domain (LBD) of ESRs to induce conformational changes of the receptors, and the ligand-receptor complexes may interact with estrogen-responsive elements (ERE) in the promoter regions of target genes and then alters the expression of target genes (Tee et al., 2004). In vertebrates, the expression levels of *ESRs* are usually closely related to hormone levels (Ogino et al., 2018). The present study found that after exposure to MET, the expression of *esr1*, *esr2a* and *esr2b* in liver decreased to varying degrees, and the expression of *ESRs* was inconsistent with hormone levels. Previous study demonstrated that basal ESRs levels might be enough to initiate oogenic protein genes (Mortensen and Arukwe, 2007). When basal *ESRs* levels are depleted, transcriptional machinery is activated to regulate the synthesis of oogenesis protein genes (Pakdel et al., 1997; Yadetie et al., 1999; Bowman et al., 2002). This suggests that even though the expression levels of *ESRs* are decreased under the MET exposure, the levels of transcriptionally generated *ESRs* still meet the requirements for regulating vitellogenin production. In addition, *ESRs* are also thought to play a role in sex differentiation due to their involvement in sex steroid signaling (Ogino et al., 2018). Currently, ESRs have been shown to mediate sex reversal in zebrafish (Lu et al., 2017). However, the decreased expression of hepatic ESRs after MET exposure in this study did not affect vitellogenin production. Therefore, it can be said that MET does not affect the ovarian development of *M. albus* by inhibiting this mether of oocytes.

To further understand the possible impact of MET on sex reversal, the expressions of sex-related genes *Dmrt1*, *Foxl3* and *JNK1* have been studied. In the present study, the expression of *Dmrt1* was inhibited under MET exposure, which is similar to the result of the effect of estrogen compounds on other teleosts (Farmer and Orlando, 2012). *Dmrt1* control the formation, development and maintenance of male reproductive organs (Gao et al., 2016; Cui et al., 2017). The results showed that the male pathway is inhibited after exposure to MET. In addition, *Foxl3* and *JNK1* were considered to play a role in sex reversal in *M. albus*, *Foxl3* is thought to promote oocyte degeneration and spermatogenesis, while *JNK1* may be important for oocyte growth and differentiation (Xiao et al., 2010; Gao et al., 2016). In the present study, the significant downregulation of *foxl3* and non-significant upregulation of *JNK1* at all MET concentrations may mean that spermatogenesis is inhibited and oocyte development is promoted after MET exposure. This indicates that MET acts more like an estrogen, inhibiting testis development but promoting oocyte development.

## Conclusion

The present study shows that MET has estrogenic effects with perturbance on female *M. albus*. MET can directly affect the expression of genes on the HPG axis without affecting the level of sex hormones. The exposure of MET increased the expression of *CYP19A1b* and *CYP17*, which regulate sex hormone production in the brain, but all genes related to sex hormone secretion in the ovary were suppressed. Furthermore, sex-associated genes did not show a trend of sex reversal after MET influence.

## Data Availability

The original contributions presented in the study are included in the article/Supplementary Material, further inquiries can be directed to the corresponding author.
